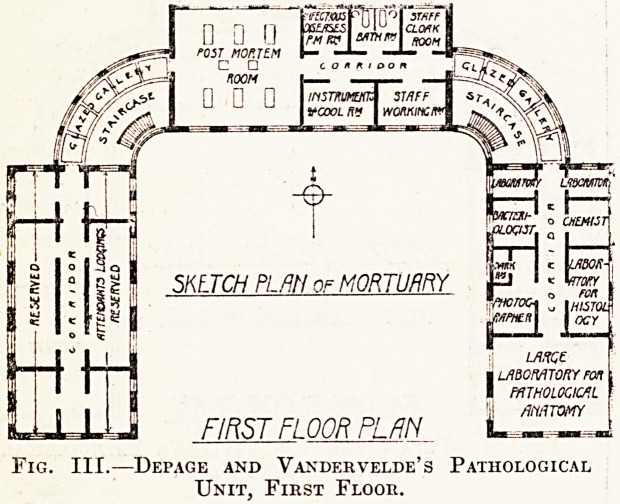# The Mortury Unit: Different Type Compared

**Published:** 1913-03-29

**Authors:** 


### THE MAKING OF A MODERN HOSPITAL.

### The Mortuary Unit: Different Types Compared.

THE INSTITUTIONAL PART.—IV. (*continued*).

We now come to the institutional part properly speaking of the mortuary unit as distinct from that part of the block which is public or semi-public. By the institutional part we mean that portion of the building to which no one who is not directly connected with the hospital staff has access without special permission from the hospital authorities. It includes the post-mortem room or rooms, the refrigerating room or chilling chamber, the laboratories, the work rooms, museum, and other offices. The number and extent of such rooms depend, of course, wholly on the size of the hospital to which the unit is attached. A large teaching hospital will, of course, have a much bigger mortuary or pathological block than a small institution which has no attached medical school. For a small hospital it may be laid down that a single post-mortem room with a small chilling chamber attached and a working room for the staff, with adequate lavatory accommodation, are all that are required. For a larger hospital, more elaboration is essential in the planning.

FIG. I.—GALTON'S MORTUARY UNIT.

The appended ground floor plan of a mortuary block for a small institution is of interest as showing the ideal of a decade ago. It provides for a public inquest room, which, as we have already tried to show, is unnecessary in most cases, but is otherwise a fairly good plan. The disposition of the doctors' room should be noted, and the arrangement of the waiting rooms at the yard end is also worth some consideration. This plan, which is taken from Sir Douglas Galton's "Healthy Hospitals," 1893, is still a very good one which deserves close study.

For purposes of comparison we give the elaborate plan of the ideal pathological block and mortuary unit designed by Depage and Vandervelde, which is given in their "Manual of Hospital Construction," 1908.

The plans explain themselves; for the measurements and details we must refer the reader to the authors' manual. One or two criticisms, however, may be made here. On the ground floor, it will be noticed, the greater part of the west wing is taken up by private mortuaries which open directly into the corridor that gives access to the waiting rooms. The chapel and undertakers' room are placed at the extremities of these private mortuaries. The east block is given over entirely to the museum and registrar's records, while the northern wing is purely institutional. This seems to us an unfair apportionment of space, since the allocation to the public part is relatively small in comparison with the large area given to the institutional portion. A feature of this design is the through corridor which connects every part of the block. Provision is made on the ground floor for an experimental class room and for a vivisection station; also for a septic

FIG. II.—DEPAGE AND VANDERVELDE'S PATHOLOGICAL UNIT, GROUND FLOOR.

autopsy room and for a store room for infectious bodies. The arrangement of the attendants' room and the adjoining attendants' bathroom on the ground floor should be noticed. On the first floor the west wing is reserved for the attendants—a wise provision—while the east wing is entirely devoted to pathological uses. The north wing is the main post-mortem room with a side room for the examination of septic cases, a conveniently arranged bathroom, and staff work rooms. The plan is from many points of view an ideal one, especially so far as the ventilation and lighting of the rooms are concerned and will well repay careful study. Its main fault is its elaborateness and the inadequate provision for the public on the ground floor. With it may be compared the plan of the new Pathological Institute at Jena, designed by Herr Peringer, in which the provision made for the post-mortem room attendant is particularly excellent, though the elaboration is even more striking, so far as the institutional portion is concerned, than in Depage and Vandervelde's plan. An interesting *via media* has been found in the planning of the mortuary block for the St. George's Hospital at Hamburg, which is one of the most interesting mortuary blocks we are acquainted with. Some interesting features are also to be found in the plans of the blocks for the new Breslau Hospital, and for the Graz surgical clinic, while in France the pathological block of the hospital at Chartres is also worth study.

### The Post-mortem Room.

As Professor Durck ably expresses it: "There is no essential reason why a modern post-mortem room should differ in arrangement and in equipment, and therefore also in cleanliness, from a modern operating theatre. Whether the operations are made upon living or dead bodies should not, in the first place, be a consideration of the architect. The more medical work is connected with the handling of dirty and infectious material, the greater should be the care that such work is done in an environment that makes it possible for it to be done without undue risk or inconvenience. After all, that is one of the first principles both of æsthetics and of hygiene, and it is difficult to comprehend that this principle is so often neglected in

Fig. III.—Depage and Vandervelde's Pathological Unit, First Floor.

the most striking manner." With these words we entirely agree. A modern post-mortem room should be as aseptic as modern arrangements can make it. By the provision of a special room for the autopsy of bodies of patients who have died of infectious diseases, it may be argued that some of this risk is avoided. But that is by no means the case. There is really in practice no reason why such a separate room should be provided, since every dead body is potentially a source of direct infection. Usually the separate room is reserved for certain special cases, the diseases reckoned as virulently infectious being smallpox, plague, and cholera. In English hospitals these diseases are rarities, and the provision of a special room to deal with them seems unnecessary, since in emergencies special provision can be made to deal with them. There is no more danger to the examining physician who conducts a post-mortem on a case of diphtheria than to one who autopsies a case of miliary tuberculosis, pyæmia, or some similar disease in which the blood is swarming with micro-organisms in a high state of pathogenic activity, provided such examination is done with proper care in a room which admits of thorough ventilation and ordinary asepsis.

One of the first essentials is that the post-mortem room must be adequately ventilated and lighted. A northern aspect is desirable in the post-mortem room, as well as in the rooms wherein microscopic work is carried on, for the simple reason that direct sunlight streaming into the room and falling on the table and the various objects to be examined may considerably interfere with the proper examination of such objects. An additional reason is that such direct sunlight may unduly raise the temperature of the rooms in midsummer so as to make the atmosphere uncomfortable for the workers. Particular care should therefore be devoted to the windows. These must be broad, with single panes of plate glass. No considerations of architectonic interest can justify the provision of small paned windows for the post-mortem rooms, or, indeed, for the whole of the mortuary block. In the rooms devoted to microscopic work due care must be taken that the parallelisms of the rays of light entering through the windows are not interfered with, as they may be by cross sections or small panes. The provision of double windows is advisable in situations where extremes of temperature are met with, and also where there is much outside noise. The simpler and more easily workable the windows are the better; in general, frames working inwards or outwards are to be preferred to sashes.

### The Best Ventilation.

The ventilation is a matter that demands close study. Perhaps the best way of ventilating the post-mortem room is by the provision of an open fireplace at both ends of the room, with floor and ceiling vents. This is, however, regarded as an old-fashioned arrangement, and is rarely to be found in newer buildings. The modern tendency is towards ventilating systems with forced draught and expulsion, sometimes aided by electrical arrangements for providing ozonised air. When the current of air entering the room is kept moist and at an even temperature—conditions which are difficult to ensure with any degree of permanency—such systems work excellently, provided always that self-registering thermometers and hygrometers are distributed in every room to permit the workers to note at once any failure of the system. Specially worthy of consideration is the high-pressure ventilation system which is employed in some operating theatres, notably at the Hamburg St. George's Hospital; this ensures a constant current of properly moistened air. The provision of ozonisers of the Siemens and Halske model is to be recommended in institutions which possess the requisite electrical motive power. Working in an ozonised atmosphere is a real pleasure to those who have had to conduct post-mortem examinations in old rooms, where the only ventilation was through window and door with wall ventilators. This subject of the proper ventilation of the working rooms of the mortuary block is one to which hospital architects should devote more attention and study.

## Figures and Tables

**Fig. I. f1:**
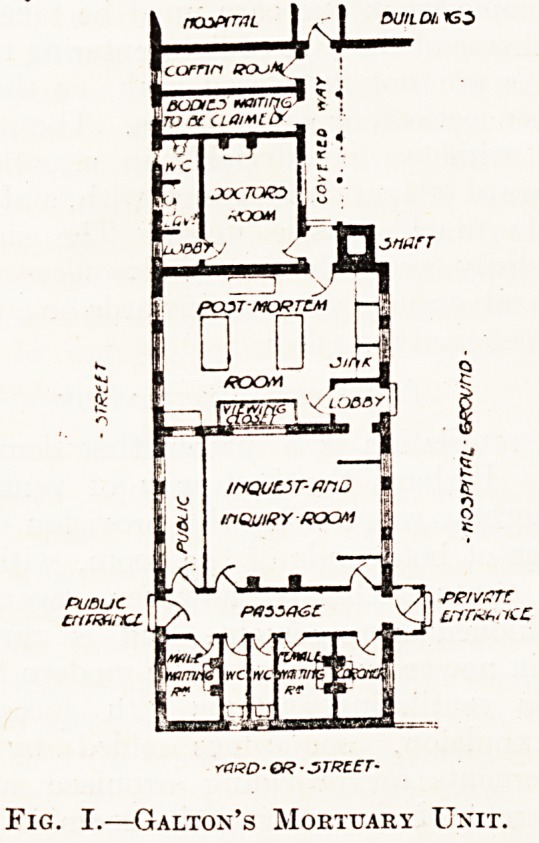


**Fig. II. f2:**
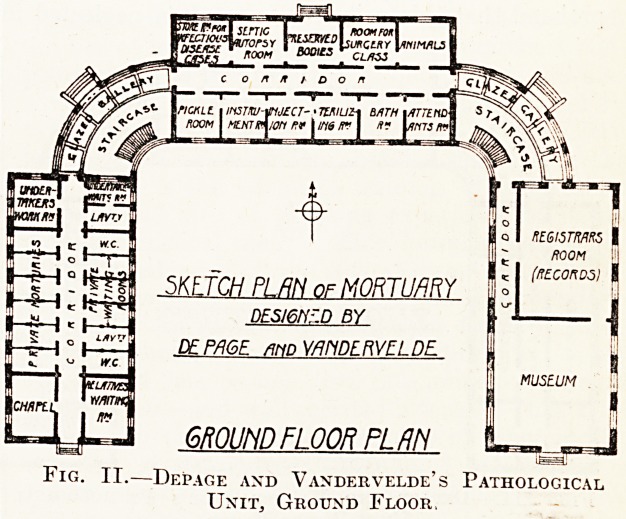


**Fig. III. f3:**